# Dual-filter IPL vs. LED phototherapy combined with systemic therapy for moderate-to-severe acne in Asian patients

**DOI:** 10.3389/fmed.2025.1742480

**Published:** 2026-01-13

**Authors:** Xuetong Zhang, Wenjun Cui, Min Chen, Min Li

**Affiliations:** 1 Department of Dermatology, The Fourth Affiliated Hospital of Soochow University, Suzhou, China; 2 Key Laboratory of Dermatology (Anhui Medical University), Ministry of Education, Hefei, China

**Keywords:** acne vulgaris, doxycycline, intense pulsed light, isotretinoin, light-emitting diode

## Abstract

**Background:**

Intense pulsed light (IPL) is a promising treatment method for acne vulgaris. However, no studies have directly compared IPL with light-emitting diode (LED) phototherapy when used as adjuncts to systemic doxycycline or isotretinoin in acne treatment.

**Objective:**

This study aimed to evaluate the efficacy and safety of IPL vs. LED phototherapy as an early intervention strategy, combined with doxycycline or isotretinoin, in patients with acne vulgaris.

**Methods:**

A prospective, split-face, controlled pilot study was conducted. Forty Asian participants with moderate-to-severe acne received a 4-week continuous split-face treatment to assess the rapid response of the two phototherapy modalities in the early phase of treatment. The protocol involved weekly IPL (400–600 nm/800–1,200 nm filter) on one side of the face and twice-weekly red-blue LED phototherapy on the contralateral side. Outcomes were assessed using the investigator-assessed Global Acne Grading System (GAGS) scores and a patient-reported Likert satisfaction scale.

**Results:**

Based on investigator assessments, IPL and LED produced comparable improvement in acne severity, demonstrating that significant improvement can be achieved early in the treatment course. In the doxycycline group, the mean improvement was 46.08% on the LED-treated side and 48.82% on the IPL-treated side; in the isotretinoin group, the mean improvement was 48.62% on the LED-treated side and 46.69% on the IPL-treated side. Patient satisfaction tended to be slightly higher on the IPL-treated side during this early stage. On the LED-treated side, “very satisfied” was reported by three participants (15%) in the doxycycline group and four participants (20%) in the isotretinoin group. Overall, there was no significant difference in efficacy between the two devices at this early time point (*p* > 0.05). In terms of safety, no serious adverse events were observed in either group. Transient adverse reactions (pain, erythema, and edema) occurred more frequently on the IPL-treated side.

**Conclusion:**

IPL is comparable to LED in clinical efficacy for the treatment of moderate-to-severe acne in Asian patients when combined with doxycycline or isotretinoin, with both modalities demonstrating rapid symptomatic improvement early in the treatment course.

**Clinical trial number:**

Identifier, ChiCTR2500107064.

## 1 Introduction

Acne vulgaris is a chronic inflammatory skin disease of the hair follicle-sebaceous gland unit, primarily presenting with open or closed comedones, papules, pustules, or nodules on the face or trunk, and may lead to pain, erythema, hyperpigmentation, or scarring (1). Global estimates of skin disease prevalence suggest that acne vulgaris is the most common skin condition worldwide. It affects up to 85% of adolescents, often begins around puberty, and may persist into adulthood. Acne has been associated with significant psychosocial burden and adverse mental health, including school absenteeism, impaired occupational functioning, and suicidal tendencies (2).

The pathophysiology of acne is multifactorial, involving follicular hyperkeratinization, microbial colonization, increased sebum production, and complex inflammatory pathways mediated by both innate and adaptive immunity. Neuroendocrine mechanisms, as well as genetic and non-genetic factors, also contribute. Moreover, clinical risk factors, such as pubertal development, family history of acne, and an oily skin type, have been associated with acne onset and severity (3). This complex interplay complicates the management of acne vulgaris.

Available treatments include topical agents, systemic antibiotics, hormonal therapies, oral isotretinoin, physical modalities, and dietary or environmental interventions. Systemic antibiotics are widely used, and doxycycline is recommended by clinical guidelines for moderate-to-severe acne (4). Since 1982, oral isotretinoin has remained the only FDA-approved systemic treatment for severe recalcitrant nodular acne vulgaris. Although its mechanism of action is not fully understood, isotretinoin reduces sebaceous gland size and secretion, indirectly decreases sebum-dependent *Cutibacterium acnes* on the skin surface and within follicles, normalizes follicular keratinization to inhibit comedogenesis, and exerts anti-inflammatory effects (4).

Physical and device-based treatments for acne include acne lesion extraction, chemical peels, laser-based and light-based devices, microneedle radiofrequency devices, and photodynamic therapy (5). Red and blue light-emitting diode (LED) therapies have been FDA-cleared since 2009 for mild-to-moderate acne vulgaris. Red light is believed to act primarily by photobiomodulation, producing anti-inflammatory effects and supporting skin repair, whereas blue light induces an endogenous photodynamic response with broad antibacterial activity against organisms such as *Cutibacterium acnes* (6). Intense pulsed light (IPL) emits polychromatic light in the range of 400–1,200 nm and may exert multiple effects, including antibacterial activity against *Cutibacterium acnes* (C. acnes), anti-inflammatory effects, suppression of sebaceous gland activity, and capillary coagulation (3). Although these approaches are standard first-line options, treatment responses are variable and rarely complete. Therefore, combination therapy is often required to optimize outcomes. Prior studies have reported that IPL combined with oral isotretinoin may be more effective than isotretinoin alone (7, 8). Photodynamic therapy combined with antibiotics has also been reported to improve clinical outcomes and quality of life (QOL) in moderate-to-severe facial acne compared with tetracyclines alone (9).

In routine clinical practice, when formulating adjunctive light-based regimens for participants with moderate-to-severe acne, clinicians often face a key therapeutic choice between systemic doxycycline, which typically provides more rapid anti-inflammatory effects, and oral isotretinoin, which offers broader disease-modifying activity but may have a slower onset of clinical response. To reflect this real-world decision-making scenario, we sought to examine the incremental value of light-based therapy under these two distinct systemic-treatment backgrounds. Therefore, in this study, we evaluated the safety and efficacy of IPL vs. LED phototherapy for moderate-to-severe acne vulgaris in Asian patients receiving oral doxycycline or isotretinoin, and explored whether comparative effectiveness and tolerability differed by concomitant systemic therapy.

## 2 Materials and methods

### 2.1 Study design

This prospective, split-face, controlled pilot study was approved by the Human Ethics Committee and conducted in accordance with the Declaration of Helsinki. Written informed consent was obtained from all participants before enrollment. The study was conducted at the outpatient dermatology department in August 2025.

### 2.2 Patient selection

A total of 40 subjects (14 men and 26 women) aged above 18 years with Fitzpatrick skin types II–V and moderate-to-severe acne (GAGS score > 20) were recruited. Since no prior comparative data were available, this study was designed as an exploratory pilot study with a small sample size. Therefore, the sample size was determined based on feasibility rather than a formal power analysis.

The inclusion criteria were as follows: (1) age ≥18 years; (2) a diagnosis of moderate-to-severe acne vulgaris defined by a Global Acne Grading System (GAGS) score >20; and (3) signed informed consent and willingness to comply with the study protocol and follow-up schedule. Exclusion criteria were as follows: use of oral isotretinoin or tetracycline-class antibiotics, or receipt of skin resurfacing therapies within the preceding 6 months; pregnancy or lactation; photosensitivity; other pre-existing dermatologic conditions that could interfere with assessments; or uncontrolled systemic diseases. Participants were instructed to avoid other cosmetic and esthetic procedures during the study period.

### 2.3 Treatment protocols

The subjects were randomly divided into two groups.

Participants were randomly assigned to one of two systemic-therapy groups. Randomization was performed using a random number table. Regarding blinding, the investigator responsible for assessing clinical improvement was blinded to the light-based treatment allocation (IPL vs. LED). The assessor evaluated standardized pre- and post-treatment photographs and performed lesion counts without knowledge of which side received IPL or LED. To support masking, photographs were obtained in a standardized manner by an independent third party, with no visible cues indicating the device used. In addition, the presentation order of photographs was randomized during data analysis. Complete blinding of participants was challenging because IPL and LED devices differ in appearance and in the sensory experience during treatment (e.g., light perception and sound). To minimize expectation bias, all participants received standardized information stating that both modalities are effective light-based treatments for acne, without a detailed explanation of their differences. The operator, delivering light-based treatments, was necessarily aware of the device used to set treatment parameters, but he did not participate in any efficacy assessments.

**Figure 1:**
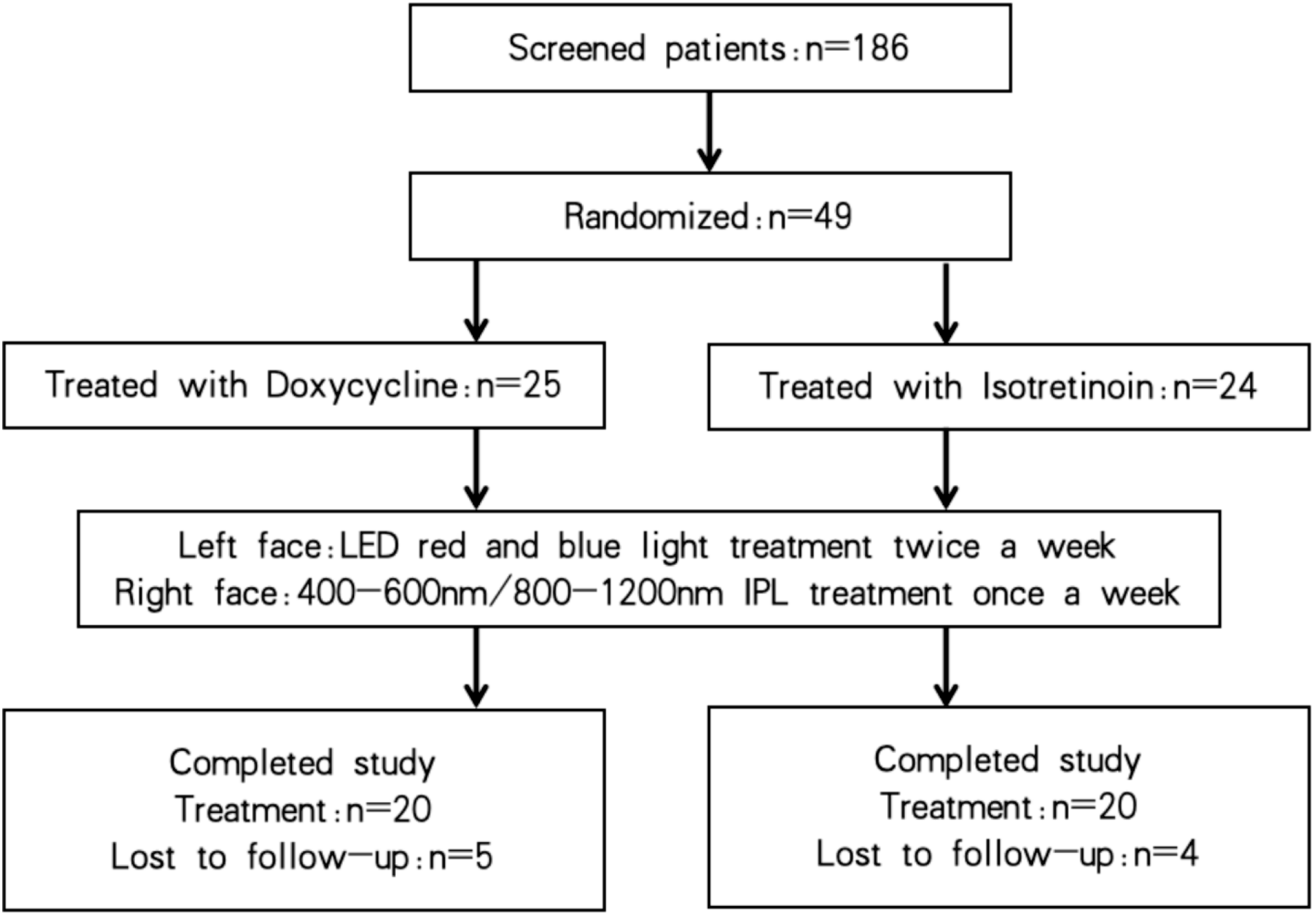
Participant flow diagram.

Group A received oral doxycycline 100 mg twice daily (Dequan Pharmaceutical, Jiangsu, China), while Group B received oral isotretinoin capsules at 0.5–0.75 mg/kg/day (Donghai Pharmaceutical, Shanghai, China). The isotretinoin dose was adjusted according to adverse events such as skin irritation. Concurrently, all participants underwent split-face light-based treatment for 4 weeks: red-blue LED phototherapy twice weekly on the left face and acne-filter IPL (400–600 nm/800–1,200 nm) once weekly on the right side. Follow-up assessments were performed 1 week after the final treatment session (Figure 1).

Participants were instructed to remove all makeup and cleanse their faces before each session. During treatment, both the participant and the treating physician wore protective goggles. For IPL treatment, a coupling cooling gel was applied before irradiation. IPL was delivered using the M22™ system (Lumenis Co., Santa Clara, CA, United States) with dual-band “acne” filters (400–600 nm and 800–1,200 nm). The spot size is 10 × 20 mm. Treatments were performed in dual-pulse mode with a pulse width of 3.5–4.0 ms and an inter-pulse delay of 30–40 ms. The fluences of the first and second pulses were 9 and 6 J/cm^2^, respectively.

On the LED-treated side, participants received combined red and blue LED phototherapy (ML-1201 LED Light Wave Therapy Instrument, Wuhan Qizhi, Wuhan, China) twice weekly. Red light (625 ± 10 nm) and blue light (470 ± 10 nm) were delivered for 20 min per session. The irradiance was 80–100 mW/cm^2^, including 25–40 mW/cm^2^ for red light and 55–60 mW/cm^2^ for blue light.

Treatment parameters were adjusted according to skin type, acne severity, pain level, and cutaneous responses during treatment. The clinical endpoint of light-based treatment was mild-to-moderate erythema and/or visible darkening of acne lesions. After each session, the treated area was immediately cooled using an ice pack for 20 min.

### 2.4 Evaluation

A detailed medical history and physical examination were performed for all participants at baseline. Treatment efficacy and safety were assessed at each visit. Standardized VISIA photographs (VISIA CRR; Canfield Scientific, Parsippany, NJ, United States) were obtained at baseline and at the final visit (front, left, and right views) for documentation and analysis.

#### 2.4.1 Efficacy

Investigator assessment (photographs). Acne severity was assessed using standardized photographs by two board-certified dermatologists who were not involved in treatment delivery. Assessments were performed using the method of the Global Acne Grading System (GAGS), as originally described by Doshi et al. GAGS divides the involved areas into six regions: forehead (factor 2), right cheek (2), left cheek (2), nose (1), chin (1), and chest/upper back (3). The factor scores for different regions are shown in parentheses. Each region is assigned a lesion grade (0 = none; 1 = ≥ 1 comedone; 2 = ≥ 1 papule; 3 = ≥ 1 pustule; 4 = ≥ 1 nodule). The regional score is calculated as factor x grade, and the total GAGS score is the sum of all regional scores. Based on the score, acne severity is categorized as mild (1–18), moderate (19–30), severe (31–38), and very severe (>39).

To accommodate the split-face design, severity and treatment response were evaluated separately for each facial side when applicable (e.g., cheek region and side-specific lesion counts), and comparisons were made between the IPL-treated and LED-treated sides.

Lesion counts and improvement rate. Acne lesion improvement for each side was calculated as improvement rate (%) = (lesion count at baseline – lesion count at follow-up)/lesion count at baseline × 100%.

Global efficacy category. Overall clinical response was categorized according to A. R. Shalita’s criteria (e.g., cured, markedly effective, effective, or ineffective). The effective rate was calculated as: effective rate (%) = (number cured + number markedly effective + number effective) / total number of participants in the group × 100%.

Participant-reported satisfaction. Participants rated their satisfaction with acne improvement using a 5-point Likert scale (1 = very dissatisfied, 2 = dissatisfied, 3 = slightly satisfied, 4 = satisfied, and 5 = very satisfied).

#### 2.4.2 Adverse events

Participants were assessed immediately after each session for treatment-related adverse events, including pain, erythema, and edema. Pain intensity was recorded using a visual analog scale (VAS) from 0 (no pain) to 10 (unbearable pain). All adverse-event assessments and recordings were performed by the same investigator, who did not deliver the light-based treatments.

At each visit, participants were also monitored for systemic adverse effects related to oral medications. Monitoring included symptoms such as dry lips/eyes/skin and gastrointestinal discomfort, and laboratory assessments (e.g., liver enzymes and lipid profile) when clinically indicated or per protocol.

### 2.5 Statistical analysis

All statistical analyses were performed using SPSS for Mac (version 26.0) and GraphPad Prism 9 (version 9.2.0). Continuous variables were presented as mean ± standard deviation (SD). Within-subject changes from baseline to post-treatment and between-modality comparisons (IPL-treated side vs. LED-treated side) were analyzed using a paired-samples *t*-test when data were approximately normally distributed. When the normality assumption was not met, the Wilcoxon matched-pairs signed-rank test was applied. Participant-reported satisfaction scores (Likert scale) were analyzed using the Wilcoxon matched-pairs signed-rank test. All tests were two-tailed, and a *p* value < 0.05 was considered statistically significant.

## 3 Results

### 3.1 Patient characteristics

Of the 186 participants screened, 49 met the eligibility criteria. Participants were randomized into two groups. During the treatment period, nine participants withdrew because of scheduling conflicts or job relocation, but none withdrew due to treatment-related adverse events. The remaining 40 participants completed the study and a 1-week post-treatment follow-up. At baseline, there were no significant between-group differences in mean age, sex distribution, skin type, or acne severity (GAGS score). Baseline demographic and clinical characteristics are summarized in Table 1.

**Table 1:** Baseline characteristics.

Parameter	Isotretinoin group (*n* = 20)	Doxycycline group (*n* = 20)	*p*-value
Characteristics			
Age (year)	24.45 ± 5.24	23.75 ± 3.32	*p =* 0.62
BMI (kg/m^2^)	20.94 ± 1.89	19.76 ± 2.53	*p =* 0.10
Gender, *n*			
Female	9	5	*p =* 0.19
Male	11	15	
Fitzpatrick skin type, *n* (II/ III/ IV/ V Type)	0/5/15/0	0/5/15/0	*p =* 1.00
GAGS grade, *n* (moderate/severe)	20/0	20/0	*p =* 1.00

The isotretinoin group comprised 9 females and 11 males, while the doxycycline group comprised 5 females and 15 males. The average age was 24.45 ± 5.24 years (range, 19–42 years) in the isotretinoin group and 23.75 ± 3.32 years (range, 18–32 years) in the doxycycline group. The average BMI was 20.94 ± 1.89 kg/m^2^ (range, 16.68–26.82 kg/m^2^) in the isotretinoin group and 19.76 ± 2.53 kg/m^2^ (range, 15.85–25.69 kg/m^2^) in the doxycycline group. Overall, Fitzpatrick skin types were III (*n* = 10, 25%) and IV (*n* = 30, 75%), with identical distribution between groups. All participants were classified as having moderate acne by GAGS grade (moderate/severe: 20/0 in each group). Baseline demographic and clinical characteristics did not differ significantly between groups (Table 1).

### 3.2 Assessment of efficacy

#### 3.2.1 Comparison of clinical improvement between LED and IPL

**Figure 2:**
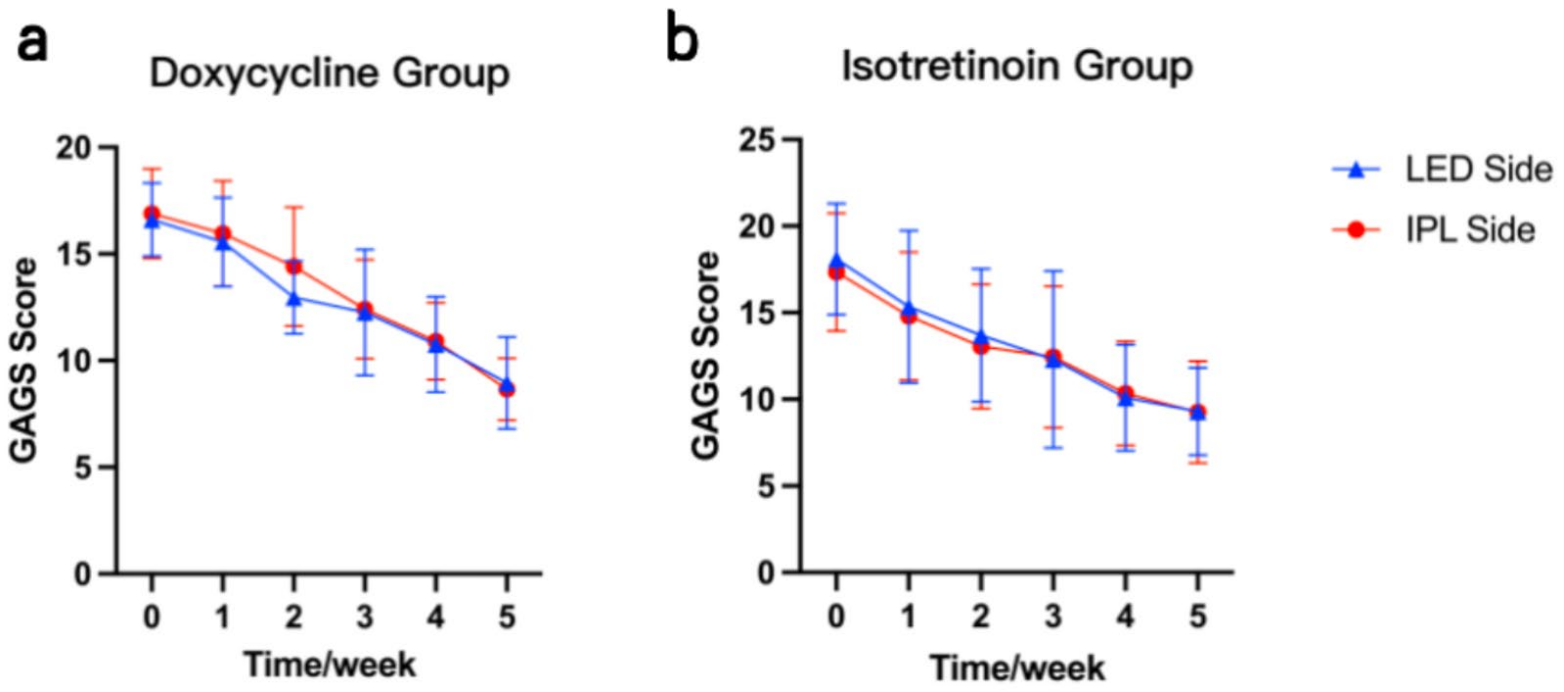
Mean (± SD) GAGS scores on the LED- and IPL-treated sides over time: **(a)** doxycycline group and **(b)** isotretinoin group.

Changes in GAGS scores from baseline to the final follow-up are presented in Table 2. After 4 weeks of treatments, both light-based modalities significantly improved acne severity (Figure 2).

**Table 2:** Mean GAGS scores.

GAGS score	LED side	IPL side	*p*-value
Isotretinoin group			*p* = 0.57
Baseline	18.10 ± 3.21	17.35 ± 3.41	
Week 5	9.30 ± 2.52	9.25 ± 2.95	
*p*-value	*p* < 0.0001****	*p* < 0.0001****	
Doxycycline group			*p* = 0.21
Baseline	16.60 ± 1.73	16.90 ± 2.10	
Week 5	8.95 ± 2.16	8.65 ± 1.46	
*p*-value	*p* < 0.0001****	*p* < 0.0001****	
Isotretinoin group vs. Doxycycline group	*p* = 0.45	*p* = 0.39	

Based on GAGS scores, clinical improvement on the LED-treated side was comparable to that on the IPL-treated side. In the isotretinoin group, mean GAGS scores decreased from 18.10 ± 3.21 at baseline to 9.30 ± 2.52 at the final visit on the LED-treated side (*p* < 0.0001****), corresponding to a mean reduction of 48.62%. On the IPL-treated side, mean GAGS scores decreased from 17.35 ± 3.41 to 9.25 ± 2.95 (*p* < 0.0001****), corresponding to a mean reduction of 46.69%. No significant difference was observed between sides at the final visit (*p* = 0.57). Similarly, in the doxycycline group, mean GAGS scores decreased from 16.60 ± 1.73 at baseline to 8.95 ± 2.16 at the final visit on the LED-treated side (*p* < 0.0001****), corresponding to a mean reduction of 46.08%. On the IPL-treated side, mean GAGS scores decreased from 16.90 ± 2.10 to 8.65 ± 1.46 (*p* < 0.0001****), corresponding to a mean reduction of 48.82%. The between-side difference was not statistically significant (*p* = 0.21).

**Figure 3:**
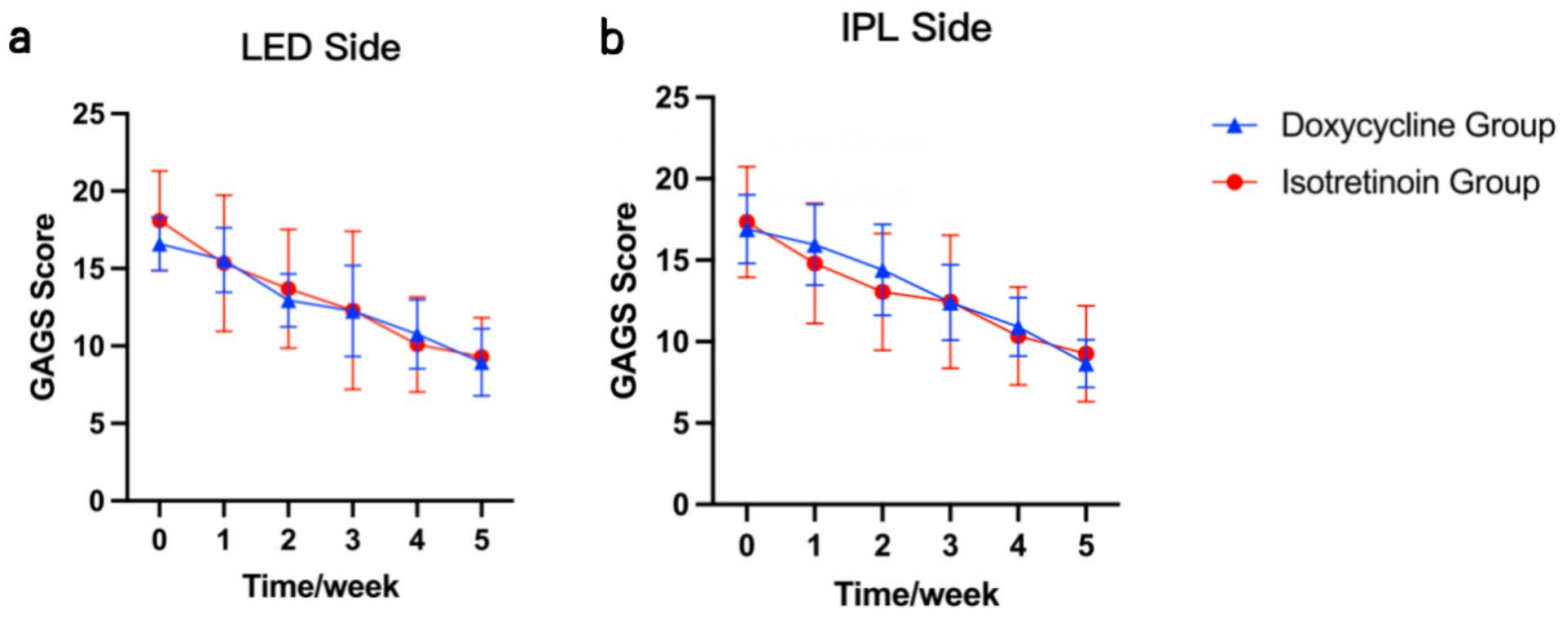
Mean (± SD) GAGS scores over time by systemic therapy group: **(a)** LED-treated side and **(b)** IPL-treated side.

On the LED-treated side, both doxycycline and isotretinoin were associated with marked improvements in acne severity with decreased GAGS scores over time, and no significant difference was observed between the two groups (*p* = 0.45). Similarly, on the IPL-treated side, improvements were observed in both systemic-therapy groups, with no significant between-group difference (*p* = 0.39). These trends are illustrated in Figure 3.

#### 3.2.2 Categorical response rates based on A. R. Shalita criteria

According to the A. R. Shalita criteria, in the isotretinoin group, the LED-treated side yielded the following: 0 cases cured, 10 markedly effective cases, 6 effective cases, and 4 improved cases, corresponding to an overall effective rate of 80%. On the IPL-treated side, there were 1 cured case, 9 markedly effective cases, 8 effective cases, and 2 improved cases, corresponding to an overall effective rate of 90%.

In the doxycycline group, the LED-treated side yielded the following: 0 cured cases, 6 markedly effective cases, 12 effective cases, and 2 improved cases, corresponding to an overall effective rate of 90%. On the IPL-treated side, there were 0 cured cases, 14 markedly effective cases, 6 effective cases, and 0 improved cases, corresponding to an overall effective rate of 100%.

**Figure 4:**
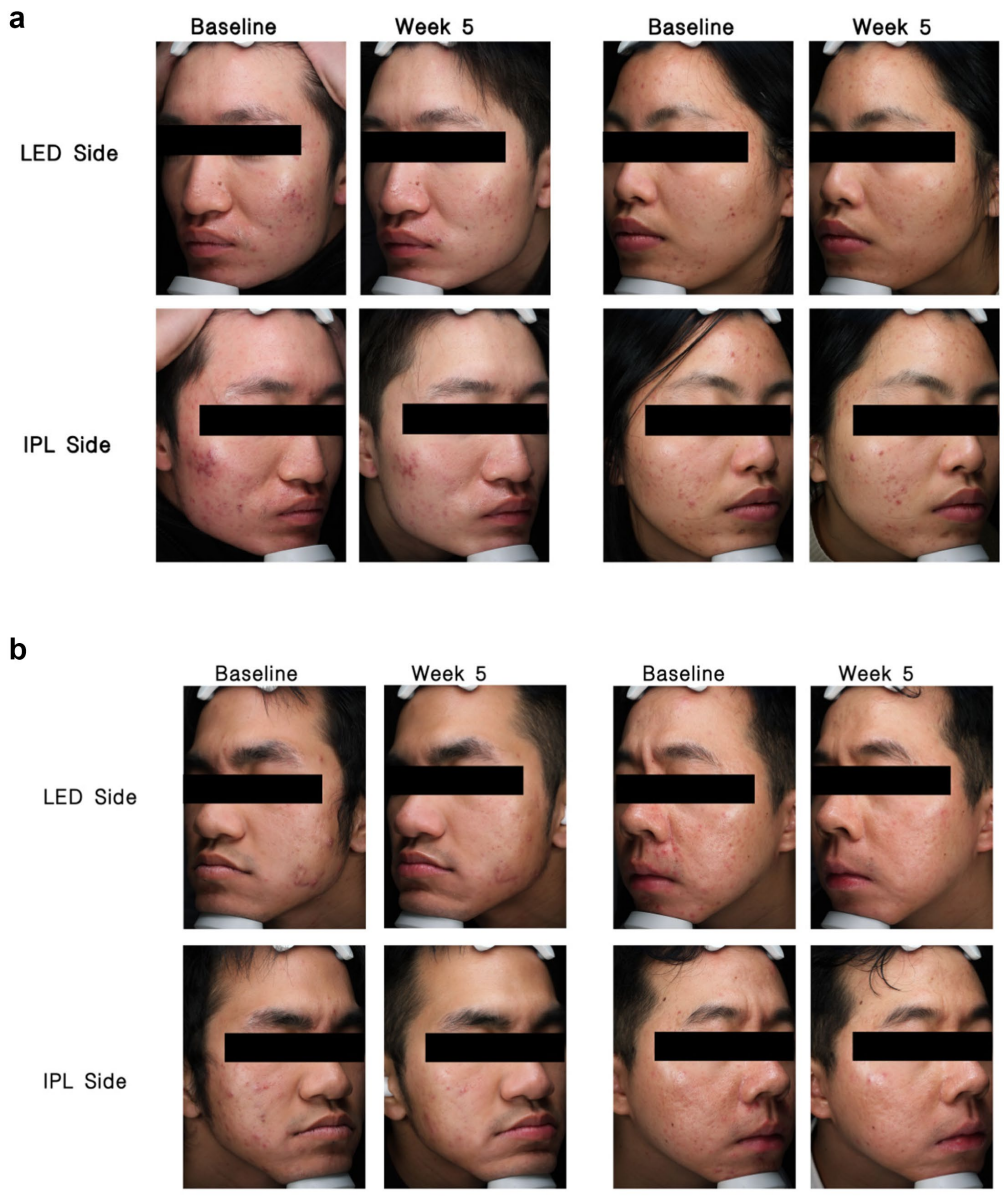
**(a)** Representative VISIA photographs from the doxycycline group at baseline and at week 5. **(b)** Representative VISIA photographs from the isotretinoin group at baseline and at week 5.

Overall, categorical response rates did not differ significantly between the LED- and IPL-treated sides (Table 3). Representative clinical photographs are shown in Figure 4.

**Table 3:** Effective response rate based on the A. R. Shalita criteria in the isotretinoin and doxycycline groups (LED vs. IPL sides).

Group	Isotretinoin group	Doxycycline group	*p*-value
LED Side	16/20(80%)	18/20(90%)	0.77
IPL Side	18/20(90%)	20/20(100%)	0.50
*P*-Value	0.76	0.43	

#### 3.2.3 Patient satisfaction

**Figure 5:**
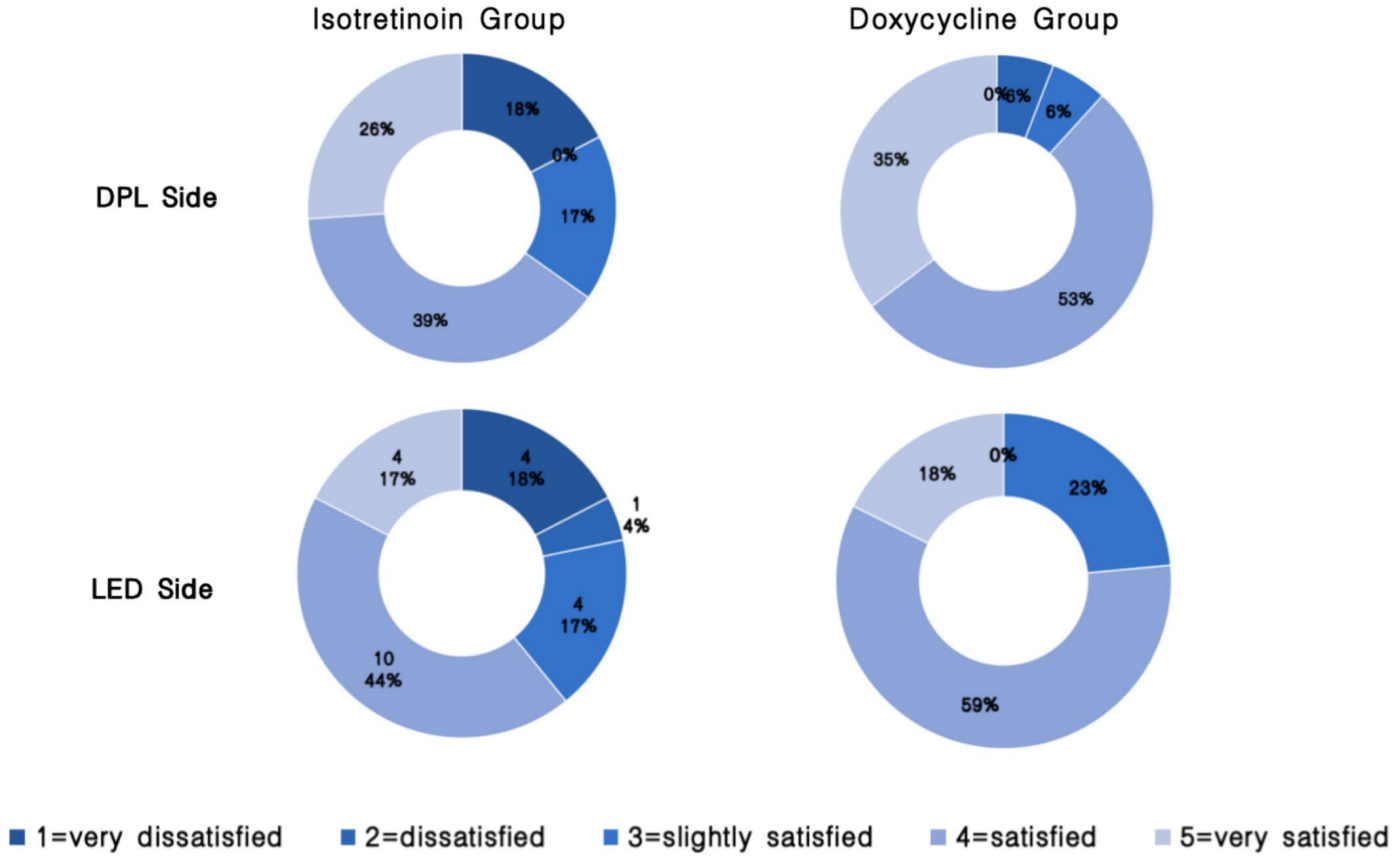
Participant-reported satisfaction with acne improvement (Likert scale) by systemic therapy group and treatment side.

Participant satisfaction ratings are shown in Figure 5. The proportion of participants reporting “very satisfied” (score 5) was higher on the IPL-treated side than on the LED-treated side in both systemic-therapy groups. However, when comparing overall Likert score distributions between sides, no statistically significant difference was observed in either the isotretinoin group (*p* > 0.99) or the doxycycline group (*p* > 0.99). Similarly, in the doxycycline group, three participants (15%) were “very satisfied” on the LED-treated side vs. six participants (30%) on the IPL-treated side (*p* > 0.99).

### 3.3 Assessment of safety

No severe adverse events were reported during the study period. No treatment-related adverse events (pain, erythema, or edema) were observed on the LED-treated side in either group. In contrast, transient local reactions were observed on the IPL-treated side, including pain, erythema, and edema.

The mean Visual Analog Scale (VAS) pain score on the IPL-treated side was 3.55 ± 0.69 in the doxycycline group and 3.74 ± 1.55 in the isotretinoin group, with no significant between-group difference (*p* = 0.50). Transient erythema on the IPL-treated side was observed in 17 of 20 participants in the doxycycline group and 18 out of 20 participants in the isotretinoin group (*p* = 0.64). Edema occurred in 4 out of 20 participants in the doxycycline group and 3 out of 20 participants in the isotretinoin group (*p* = 0.69; Table 4).

**Table 4:** Adverse events on the IPL-treated side by systemic therapy group.

Adverse event	Doxycycline group	Isotretinoin group	*p*-value
Pain	3.55 ± 0.69	3.74 ± 1.15	*p* = 0.50
Erythema, n	17/20	18/20	*p* = 0.64
Edema, n	4/20	3/20	*p* = 0.69

During systemic therapy, participants were monitored for mucocutaneous dryness (dry mouth, dry eyes, and facial skin dryness), laboratory abnormalities (liver enzymes and cholesterol), and gastrointestinal adverse events. Dryness-related adverse events were more frequent in the isotretinoin group than in the doxycycline group (12/20 vs. 3/20, *p* < 0.01). Gastrointestinal adverse events were reported by two participants in the doxycycline group, whereas none were reported in the isotretinoin group. No participants in either group exhibited elevated liver enzymes or increased cholesterol levels during the treatment period (Table 5).

**Table 5:** Drug-related adverse events by systemic therapy group.

Adverse events	Doxycycline group	Isotretinoin group	*p*-value
Xerostomia/xerophthalmia/Facial skin dryness	3/20	12/20	*p* < 0.01
Liver enzyme elevation	0	0	NA
Cholesterol elevation	0	0	NA
Gastrointestinal adverse events	2	0	NA

## 4 Discussion and conclusion

In this prospective split-face, controlled pilot study, both red-blue LED phototherapy and acne-filter IPL led to significant improvements in acne severity. Participant-reported satisfaction tended to be higher on the IPL-treated side, whereas investigator-assessed outcomes did not show a statistically significant difference between modalities. Overall, our findings suggest that LED and IPL provide comparable clinical efficacy when used as adjuncts to systemic doxycycline or isotretinoin in Asian patients. In terms of safety, no serious adverse events were observed. No clinically significant treatment-related adverse events were noted on the LED-treated side. On the IPL-treated side, transient local reactions, such as pain, erythema, and edema, were observed, consistent with the expected tolerability profile of IPL. To our knowledge, this is the first split-face clinical study to directly compare LED and IPL as adjuncts to doxycycline or isotretinoin for moderate acne in the Asian population.

Systemic antimicrobial therapy remains a cornerstone in the management of moderate-to-severe acne. Doxycycline, a second-generation tetracycline with broad antimicrobial activity, is known to achieve therapeutic concentrations within pilosebaceous units and also exerts anti-inflammatory effects, including inhibition of neutrophil chemotaxis and modulation of inflammatory mediators (10).

Isotretinoin is effective in acne largely by reducing sebaceous gland activity and sebum production, normalizing follicular keratinization, and exerting downstream effects on *Cutibacterium acnes* colonization via retinoid receptor-mediated pathways. In addition, isotretinoin can promote cell-cycle arrest and apoptosis in sebaceous cells. Mechanistically, FOXO1 has been implicated in sebaceous cell apoptosis and the inhibition of keratinocyte proliferation. Furthermore, isotretinoin also has anti-inflammatory effects due to reduced monocyte Toll-like receptor-2 expression, inhibition of neutrophil and monocyte chemotaxis, and reduced levels of pro-matrix metalloproteinase (MMP)-9 and other matrix metalloproteinases (11). IPL has become a non-invasive method for acne treatment with minimal adverse events. A substantial body of evidence indicates that IPL is very effective and safe in treating acne vulgaris, as well as post-acne erythema and hyperpigmentation. The M22 acne filter has a wavelength range of 400–600/800–1,200 nm. The acne filter has certain advantages for patients with inflammatory papules. The peak absorption of porphyrins occurs at 400–600 nm, which can lead to the generation of singlet oxygen through photo-oxidation after IPL treatment, thereby eliminating *Cutibacterium acnes*. The 800–1,200 nm range can inhibit the sebaceous glands and reduce lipid secretion (10).

LED therapy can increase ATP production, regulate intracellular oxidative stress, induce transcription factors, alter collagen synthesis, stimulate angiogenesis, and enhance blood flow. Red light can reach the dermis, activate fibroblasts, and increase the expression of fibroblast-related factors, such as type I procollagen and matrix metalloproteinase-9 (MMP-9). Blue light has lower penetration but may be useful for improving conditions in the epidermal layer (12).

Studies have shown that the use of IPL devices with the ACNE filter (400–600 nm/800–1,200 nm) alone has a significant therapeutic effect on acne vulgaris without causing significant adverse events, such as pain, erythema, edema, folliculitis, crusting, or exfoliation (13). The combined treatment of minocycline and IPL with the 400–600/800–1,200 nm acne filter can improve inflammatory lesions and the severity of acne vulgaris better than treatment with minocycline alone. Compared to minocycline treatment alone, the combined treatment also offers other advantages, such as reducing post-acne erythema and hyperpigmentation. Moreover, the adverse reactions caused by IPL are manageable (10). Delicate pulsed light (DPL) combined therapy accelerated the treatment effect of isotretinoin used alone for moderate-to-severe acne. The combined treatment is safe, with no serious adverse events reported; only minor side effects were observed (8). LED therapy has been proposed as a more affordable alternative for acne treatment. In a meta-analysis, all relevant studies showed strong homogeneity and high statistical significance, and the results confirmed that blue and red LED therapies are highly effective in treating acne vulgaris, achieving outstanding results, including a significant reduction in inflammatory and non-inflammatory acne lesions (14). However, there is still debate regarding whether there are differences in clinical efficacy between IPL and LED treatments.

In this study, in both the doxycycline group and the isotretinoin group, LED showed comparable clinical efficacy to IPL, with fewer adverse events such as pain, erythema, and edema. From a cost perspective, LED may represent a more cost-effective option. During follow-up, we noted that participant-reported satisfaction was higher on the IPL-treated side. For nodulocystic lesions with a diameter > 0.5 cm, a single IPL session appeared to produce a better therapeutic response. We speculate that this may be related to IPL’s ability, compared with LED, to deliver short, high-power pulses in the 800–1,200 nm range, which may more effectively target sebaceous glands, reduce sebaceous gland size, and decrease sebum secretion (15, 16). Within the short treatment course, both oral doxycycline and isotretinoin significantly improved clinical acne symptoms, with no significant difference between the two systemic regimens. As an exploratory pilot study, our findings are intended to inform the design of future randomized controlled trials with longer treatment durations (e.g., 12–24 weeks). In addition, this study compared two treatment strategies rather than directly comparing the two systemic drugs. In our design, systemic therapy served as a “baseline platform,” and the core objective was to evaluate the incremental value of IPL vs. LED on top of this platform. Although this approach cannot quantify the absolute contribution of light-based therapy alone, the results provide practical guidance for device selection when combination therapy has already been chosen in routine clinical practice.

In addition to clinical response, the risk of side effects is another factor that needs to be considered in practice. We are aware of long-term concerns regarding photosensitivity when oral medications are combined with light-based therapies. Minocycline has a peak absorbance at 348 nm, but minimal absorbance in the 400 to 450 nm range, and negligible absorbance at wavelengths above 450 nm (10). The photosensitivity associated with tetracycline-class antibiotics primarily occurs within the 325 to 425 nm range. Notably, the photoactivation mechanism of tetracycline derivatives does not overlap with the spectral range typically employed in IPL therapy. Therefore, in clinical practice, the use of IPL devices equipped with a cut-off filter (cut-off wavelength >400 nm) in combination with anti-inflammatory doses of sustained-release doxycycline does not appear to significantly increase the risk of photosensitivity-related adverse events (17). Isotretinoin has a peak absorbance in the UVB range, specifically around 340 nm. It exhibits higher absorbance in the UVB region (280–320 nm) and lower absorbance as the wavelength increases into the UVA region (320–400 nm). Accordingly, the ACNE filter used in this study (400–600/800–1,200 nm) and the red and blue LED wavelengths (625 ± 10 nm and 470 ± 10 nm) are outside the primary absorption ranges implicated in phototoxicity associated with minocycline or isotretinoin. In addition, IPL and LED treatments were administered for only 4 weeks. Therefore, any treatment-related adverse events would be expected to be transient and mild. Consistent with this, we observed no serious adverse events during the treatment period.

Common phototherapy-related adverse events, such as pain, erythema, and edema, were not observed on the LED-treated side in either group. On the IPL-treated side, participants experienced varying degrees of pain, erythema, and edema. However, these reactions were alleviated after ice-pack cooling and were not related to the choice of oral medication.

During oral isotretinoin therapy, 60% of participants experienced dryness-related adverse events, including dry mouth, dry eyes, and facial skin dryness, compared with 15% in the doxycycline group. We speculate that this difference is related to isotretinoin’s sebum-suppressive mechanism, and symptoms improved after appropriate dose reduction. In addition, 10% of participants reported gastrointestinal adverse events while taking oral doxycycline, although these were tolerable. Importantly, no participants developed elevated liver enzymes or increased cholesterol levels during the treatment period, which are key laboratory abnormalities requiring monitoring.

This study has several limitations. First, all participants had a similar racial background, which may limit the generalizability of the findings to other populations. Second, due to the lack of prior comparative data, we were unable to perform a formal sample size calculation. Therefore, we conducted an exploratory pilot study with a relatively small sample size. The sample size was determined based on feasibility considerations rather than a power analysis, and future studies will require larger cohorts. Third, the follow-up period was short and should be extended to assess long-term outcomes.

As an exploratory study, our results are intended to inform the design of future randomized controlled trials with longer treatment durations (e.g., 12–24 weeks). Importantly, the primary goal of the present study was not to evaluate the final cure rate of acne, but to quantify and compare the early-onset effects of two light-based devices (dual-filter IPL vs. LED) when used in combination with systemic therapy. In clinical practice, both patients and clinicians value the speed of response, as it directly affects treatment adherence and early improvements in quality of life. The 4-week time point represents a critical window to determine whether adjunctive phototherapy can “accelerate” clinical improvement. Mechanistically, the core advantages of IPL and LED phototherapy include rapid anti-inflammatory and antibacterial effects, which are typically most evident in the early phase of treatment. Therefore, selecting a 4-week observation period was intended to best capture and quantify this “early anti-inflammatory benefit” of adjunctive phototherapy. Finally, we fully acknowledge that the current design limits the interpretation of the independent effect of phototherapy. In future work, we plan to conduct a more rigorous three-arm randomized controlled trial to more precisely quantify the independent contribution of each phototherapy modality.

## 5 Conclusion

Overall, both IPL and LED phototherapy were effective in improving acne. Based on investigator assessments, IPL did not demonstrate a significant advantage over LED for moderate-to-severe acne when used concurrently with isotretinoin or doxycycline. Participant-reported satisfaction tended to be higher on the IPL-treated side, but the differences were not statistically significant. Given the higher frequency of transient local adverse events on the IPL-treated side (e.g., erythema, edema, and pain), LED may be considered a more tolerable option for Asian patients receiving adjunctive light-based therapy.

## Data Availability

The original contributions presented in the study are included in the article/supplementary material, further inquiries can be directed to the corresponding author.
